# Trem2 negatively regulates the MTOR-PKCα axis to protect against *Toxoplasma gondii*-induced adverse pregnancy outcomes

**DOI:** 10.3389/fcimb.2026.1897580

**Published:** 2026-07-30

**Authors:** Xiaoyu Geng, Yangchenxi Lu, Xinyi Zhou, Xiaoyao Xu, Yahan Liu, Junfang Zhang, Jinling Chen

**Affiliations:** 1 Department of Pathogen Biology, School of Medicine, Nantong University, Nantong, China; 2 Eye Institute, Affiliated Hospital of Nantong University, Medical School of Nantong University, Nantong, China

**Keywords:** adverse pregnancy outcomes, mTOR, PKCα, *Toxoplasma gondii*, Trem2

## Abstract

**Background:**

Triggering receptor expressed on myeloid cells 2 (Trem2) deficiency aggravated adverse pregnancy outcomes caused by *Toxoplasma gondii* (*T. gondii*) infection during pregnancy; however, the precise molecular mechanisms involved remain to be fully elucidated.

**Methods:**

Molecular docking, co-immunoprecipitation, and alanine scanning mutagenesis were employed to identify the interaction between Trem2 and mammalian target of rapamycin (MTOR). *In vivo* experiments utilized a *T. gondii*-infected pregnant mouse model with wild-type and *Trem2*-knockout mice, while *in vitro* studies were performed using Raw264.7 macrophages and bone marrow-derived macrophages (BMDMs) with *T. gondii* antigen stimulation, *Trem2* overexpression, and siRNA-mediated *MTOR* knockdown.

**Results:**

A direct high-affinity interaction between Trem2 and MTOR was identified, with GLN-198, ARG-195, and LYS-199 as critical binding residues. *T. gondii* infection significantly downregulated Trem2 expression while upregulating MTOR and its downstream effector protein kinase C alpha (PKCα). *Trem2* deficiency exacerbated MTOR-PKCα hyperactivation upon infection and aggravated placental hemorrhage, necrosis, and fetal growth restriction. Conversely, *Trem2* overexpression suppressed MTOR and PKCα expression, and *MTOR* knockdown confirmed that PKCα acts downstream of MTOR.

**Conclusion:**

Trem2 negatively regulates the MTOR-PKCα axis through direct interaction, and *T. gondii* infection downregulates Trem2 to relieve this inhibition, leading to MTOR-PKCα hyperactivation and adverse pregnancy complications.

## Graphical Abstract

**Figure:**
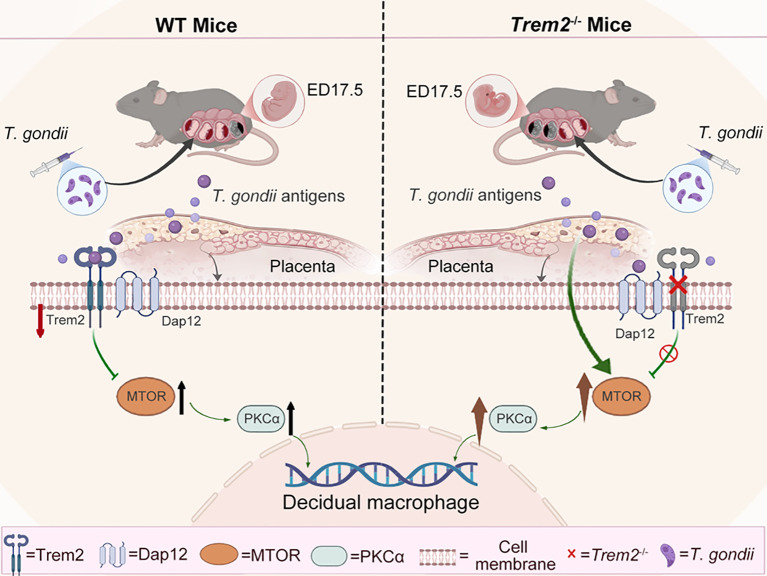

## 1 Introduction

A widely prevalent zoonotic pathogen, *Toxoplasma gondii* (*T. gondii*) poses a particularly serious threat to pregnancy health (Salari et al., 2025). During early pregnancy, the maternal body is in a physiologically immunocompromised state to maintain maternal-fetal tolerance, facilitating embryo implantation and development (April and Long, 2024; Cho et al., 2025). This state makes pregnant women susceptible to vertical transmission of the pathogen through the placenta upon infection, leading to congenital toxoplasmosis, which clinically manifests as miscarriage, fetal developmental abnormalities, or stillbirth (Silva et al., 2024; Cerisola et al., 2025). Findings indicate that *T. gondii* primarily exerts its pathogenic effects by successfully breaking down the normally sustained immune tolerance equilibrium between mother and fetus (Gao et al., 2022; Arshad et al., 2025). As the key regulatory cells at this interface, decidual macrophages control embryo implantation, induce immune tolerance, and modulate inflammation by undergoing phenotypic polarization and functional conversion (Liu and Zhang, 2025; Ma et al., 2026). Previous reports indicate that *T. gondii* directly modulates decidual macrophage activity through the secretion of various effector molecules (Guo et al., 2025; Salomão Lopes et al., 2025). For instance, the *T. gondii* dense granule protein 28 (GRA28) regulates the secretion of C-C motif chemokine ligand 22 (CCL22) from trophoblast cells via the myc regulation 1 (MYR1) axis, contributing to miscarriage (Rudzki et al., 2021). On the other hand, dense granule protein 15 (GRA15) drives the expression of pro-inflammatory cytokines (e.g., IL-1β and IL-12), triggers the NF-κB signaling cascade, and shifts macrophage polarization toward an M1-like state (Arranz-Solís et al., 2021). Concurrently, research has also found that abundant IL-10 secreted by M2-polarized decidual macrophages helps protect against adverse pregnancy complications (APC) resulting from *T. gondii* infection (Liu et al., 2018). This evidence suggests that *T. gondii* infection likely disrupts tolerance between mother and fetus by secreting effector molecules that manipulate decidual macrophage function, ultimately leading to APC. Therefore, this study sought to elucidate the key host protective molecule and its downstream signaling cascade involved in preserving decidual macrophage function during *T. gondii* infection.

Expressed broadly on myeloid cells, Trem2 (triggering receptor expressed on myeloid cells 2) is a surface receptor that critically regulates multiple cellular functions, including survival, migration, phagocytosis, and cytokine secretion (Wei et al., 2024; Wang et al., 2025; Zhu et al., 2025b). Researchers comparing decidual macrophages with peripheral blood CD14^+^ cells have identified Trem2 as one of the characteristically highly expressed genes in decidual macrophages, constituting its immunoregulatory phenotype along with M2-type markers such as CCL-18 and CD209 (Gustafsson et al., 2008). Meanwhile, single-cell transcriptomic analyses of placentas from preeclampsia cases have also revealed Trem2^+^ macrophages as major participants in the placental immune niche (Admati et al., 2023; Li et al., 2024). Importantly, Trem2 not only directly regulates macrophages but also suppresses inflammatory responses through its downstream signaling pathways (Medd, 2025). For example, Trem2 disrupts glycolysis via the JAK2/STAT3 axis, thereby inhibiting M1 polarization and inflammation (Liu and Zhou, 2025). However, this protective mechanism becomes markedly disrupted when *T. gondii* infection occurs. We previously reported that *T. gondii* infection downregulates Trem2 expression, consequently disrupting the M1/M2 balance and creating a pro-inflammatory milieu across the maternal-fetal interface (Wang et al., 2024). Emerging evidence indicates that MTOR is a key regulator of macrophage function and maternal-fetal immune homeostasis (Kramer et al., 2023). In addition, *T. gondii* can manipulate host MTOR signaling to facilitate intracellular survival (Wang et al., 2009). Nevertheless, although both Trem2 and MTOR have independently been implicated in macrophage biology, pregnancy-associated immune regulation, and *T. gondii* infection, whether Trem2 directly regulates MTOR signaling in decidual macrophages, and whether disruption of this regulatory axis contributes to APC following *T. gondii* infection, remain largely unknown.

Based on this, the present study first confirmed the potential interaction between Trem2 and MTOR through bioinformatic prediction and co-immunoprecipitation, and precisely identified the key binding sites using alanine scanning mutagenesis. Both cell-based and animal studies demonstrated that *T. gondii* infection significantly reduced Trem2 expression while upregulating MTOR and its downstream effector Protein kinase C alpha (PKCα). In summary, this study reveals for the first time that Trem2 acts as a negative regulator of MTOR, suppressing MTOR expression through direct interaction and subsequently downregulating downstream PKCα signaling. *T. gondii* infection, by downregulating Trem2, leads to hyperactivation of the MTOR-PKCα axis, ultimately driving M1/M2 imbalance in decidual macrophages and APC. This discovery provides a new understanding of immunopathological mechanisms of toxoplasmosis and lays a theoretical foundation for developing intervention strategies for *T. gondii* infection during pregnancy.

## 2 Methods

### 2.1 Mice

All experimental procedures followed the ARRIVE guidelines (Animal Research: Reporting of *In Vivo* Experiments). A CRISPR/Cas9-generated *Trem2*-knockout strain (B6/JGpt-Trem2em1Cd3332/Gpt; Gempharmatech Co., Ltd) and C57BL/6 mice were maintained at Nantong University. Timed pregnancies were established by overnight mating. Females were examined for a vaginal plug the following morning, with the detection date defined as embryonic day 0.5 (ED0.5) (Guo et al., 2023). At ED8.5, plug-positive dams were randomly allocated into two experimental groups: one received an intraperitoneal injection of sterile phosphate-buffered saline (control group, NP), while the other received 300 tachyzoites of the *T. gondii* RH strain (*T. gondii* infection group, TI). To minimize bias, the investigator performing the injections and subsequent phenotypic assessments was blinded to the group allocation throughout the experiment. In addition, data acquisition and analysis, including Western blot quantification and fluorescence intensity measurements, were performed using anonymized datasets by investigators blinded to experimental groups. The health and survival of all pregnant mice were monitored daily. Any mice that died prior to the scheduled endpoint at ED17.5 was excluded from the final dataset. Pregnancies were terminated at ED17.5 by CO_2_ euthanasia. Placentas were collected for analysis, and fetal development was quantified by measuring body weight and calculating a size index based on the product of crown-rump length and occipitofrontal diameter (CRL × OFD). The Animal Care and Use Committee of Nantong University approved all protocols (Approval No. P20230302-013).

### 2.2 Preparation of *T. gondii* antigens

Tachyzoites of the *T. gondii* RH strain was used as the source for antigen preparation (Qiu et al., 2016). Following incubation of 1×10^8^ parasites (viability >95%) in serum-free RPMI-1640, the supernatant was concentrated using Amicon^®^ Ultra-15 centrifugal filters (Merck Millipore, Germany). A specialized removal kit (Thermo Fisher Scientific, USA) was then applied to remove endotoxins prior to storage at -80 °C.

### 2.3 Isolation and culture of macrophages

Raw264.7 macrophages were obtained from the National Collection of Authenticated Cell Cultures (Shanghai) and cultured under standard conditions (Fu et al., 2022). Subculturing was performed when cell confluence reached approximately 70%-80%. Tibias and femurs of 6-8-week-old WT and *Trem2*^-/-^ mice were used to isolate BMDMs. Briefly, marrow was harvested by flushing the bones with complete medium. The cell suspension was treated with red blood cell lysis buffer, then centrifuged and filtered through a 200 µm mesh. Cells were then cultured in complete medium supplemented with murine M-CSF (20 ng/mL) to induce macrophage differentiation.

### 2.4 Immunoprecipitation

Samples were lysed in ice-cold RIPA buffer with protease inhibitors, then centrifuged. For each immunoprecipitation, equal amounts of total protein were pre-cleared by incubation with Protein A/G agarose beads (SC-2003, Santa Cruz, Dallas, USA) with gentle rotation. Pre-cleared supernatants were then exposed to an anti-MTOR antibody (1: 100, rabbit monoclonal, #C15M14, Selleck, Houston, USA) or a control IgG antibody under constant rotation. Protein A/G agarose beads were then incubated to pull down antigen-antibody complexes, collected by centrifugation. Finally, protein expression was assessed by WB.

### 2.5 Immunoblotting

Immunoblotting experiments were performed as described (Wang et al., 2020). RIPA buffer with protease inhibitors was used to extract protein from tissues or cells. The lysis buffer was prepared at a volume ratio of RIPA plus 1% PMSF and 1% protease inhibitor cocktail. Following quantification, equal protein loads were separated by SDS-PAGE using 8%-10% gels and then blotted onto PVDF membranes. Cells were kept for one night at 4°C with primary antibodies: anti-MTOR (1: 1000, Selleck), anti-Trem2 (1:1000, EPR26210-1, #ab305103, Abcam, Cambridge, UK), anti-FAK (1:2000, sc-558, Santa Cruz), anti-P38 (1:2000, 14064-1-AP, Proteintech, Rosemont, USA), anti-GAPDH (1:10000, #60004-1-Ig, CST, Danvers, USA), and anti-PKCα (1:1000, rabbit monoclonal, D7E6E, # 59754, CST). Membranes were exposed to anti-mouse (1: 5000, #SA00001-1, Proteintech) or anti-rabbit (1:5000, #BL003A, Biosharp, Jiangsu, China) secondary antibodies. Protein bands were detected using ECL substrate (Meilunbio, Dalian, China) and quantified with Image J software, normalizing to GAPDH as the loading control.

### 2.6 Immunofluorescence staining

For immunofluorescence analysis, cells were 4% PFA-fixed. Cells were permeabilized with 0.1% Triton X-100 in PBS, then blocked with 5% BSA. Subsequently, cells were kept overnight at 4°C with diluted primary antibodies, including anti-MTOR antibody (1:250, Selleck), anti-Trem2 (1:500, Abcam), and anti-PKCα (1:100, CST). Cells were washed in PBS and then exposed to suitable fluorophore-linked secondary antibodies (abs50028, Absin, Shanghai, China). DAPI was applied for 5 min to counterstain nuclei. Mounted coverslips were then visualized by confocal microscopy. After mounting the coverslips, samples were observed and imaged with a confocal microscope.

### 2.7 Overexpression constructs

To reconstitute Trem2 signaling *in vitro*, a chimeric *Trem2*-*DAP12* receptor was engineered (Zhao et al., 2022). We constructed this fusion protein by fusing mouse *Trem2*′s extracellular domain (aa 19-171) and intracellular segments of mouse *DAP12* (aa 28-114). A key aspartate-to-alanine mutation (D52A) was introduced in the *DAP12* moiety to abrogate its promiscuous interactions with other receptors, thereby ensuring the exclusive. For enhanced secretion and detection, the peptide was substituted with the mouse Igκ leader sequence, and an N-terminal 3×FLAG tag was incorporated. The corresponding lentiviral overexpression plasmid (pcSLenti-EF1-EGFP-F2A-Puro-CMV-Igκ leader-3xFLAG-*Trem2*-*Tyrobp*) and an empty vector control were packaged into viral particles by OBiO Technology (Shanghai, China). Lentiviral transduction was performed on Raw 264.7 cells at 30%-40% confluence using an MOI of 40. Following 12–16 h, the virus-containing medium was replaced with DMEM. Starting at 72 h after transduction, cells were selected with 2 µg/mL puromycin (Beyotime) for one week to obtain stable polyclonal populations. Overexpression of the fusion protein was then verified by WB.

### 2.8 RNA interference

To achieve *MTOR* knockdown, siRNAs targeting MTOR along with a negative control (siNC) were purchased from Genepharma (Jiangsu, China). Raw264.7 cells were transfected with these siRNAs using INTERFERin Reagent (Polyplus, Strasbourg, France). After 6 h of transfection, cells were further cultured for 48 h with *Tg*Ag (5 µg/mL) or culture medium alone. The siRNA sequences were used to target MTOR: Forward, 5′-CCACCAGAAUUGGCAGAUUTT-3′; Reverse, 5′-AAUCUGCCAAUUCUGGUGGTT-3′.

### 2.9 Statistical analysis

Results represent at least three independent biological replicates and are presented as mean ± SD. All statistical analyses were performed with GraphPad Prism software (version 9.0). To compare two groups, we applied an unpaired Student′s *t*-test. To compare multiple groups, we used one-way ANOVA with Tukey′s *post hoc* test for single-factor designs, and two-way ANOVA with Sidak′s test for factorial designs. Statistical significance was set at *P* < 0.05.

## 3 Results

### 3.1 Trem2 interacts with MTOR to modulate immune signaling pathways

**Figure 1:**
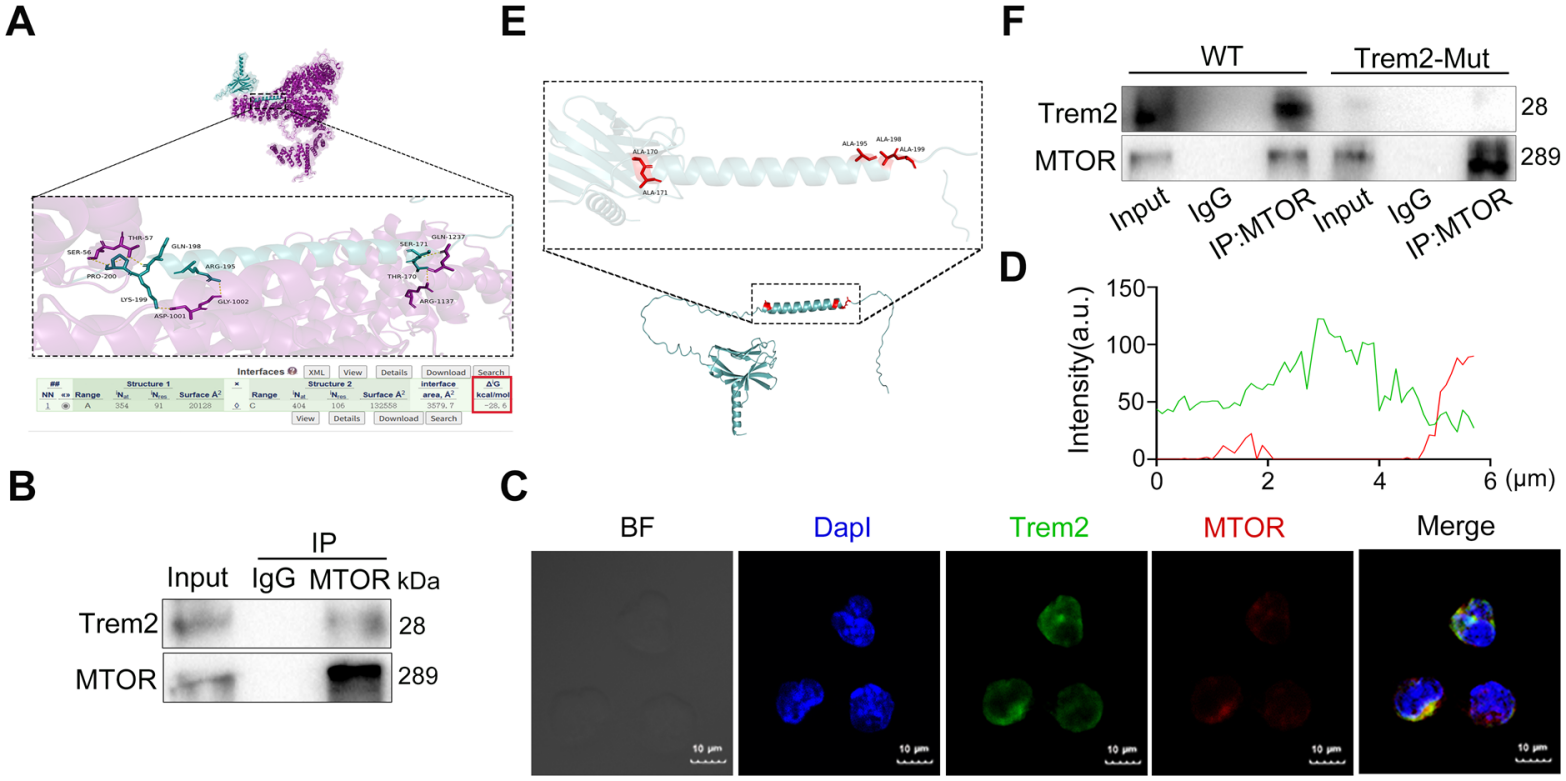
Trem2 directly interacts with MTOR. **(A)** Molecular docking analysis (PDBePISA) identified a strong interaction between Trem2 and MTOR, yielding a binding energy of -28.6 kcal/mol. Trem2-MTOR complex structure was visualized in three dimensions with PyMOL. **(B)** To confirm the interaction, cell lysates underwent immunoprecipitation using an anti-MTOR antibody, and the presence of both MTOR and Trem2 was subsequently detected by immunoblotting. **(C)** Raw264.7 cells were examined by immunofluorescence for Trem2 (green) and MTOR (red) co-localization, with nuclei counterstained by DAPI. **(D)** Co-localization and fluorescence intensity of Trem2 and MTOR were quantified with Image J. The results are expressed in arbitrary units (a.u.). **(E)** Based on the molecular docking model, five predicted interface residues (GLN-198, ARG-195, LYS-199, SER-171, and THR-170) were individually mutated to alanine. **(F)** Wild-type or Trem2 mutant constructs were co-expressed with MTOR in HEK293T cells, and the interaction was assessed by immunoprecipitation.

To explore the downstream signaling mechanisms of Trem2, we sought to identify its potential interacting molecules. *T. gondii* infection has been reported to activate the MTORC1/2 pathway in trophoblast cells, leading to upregulated MTOR expression (Leroux et al., 2018). Furthermore, MTOR hyperactivation has been observed in decidual tissues of patients with recurrent pregnancy loss as well as in relevant mouse models. This raises the possibility that excessive MTOR activation could be a central link between *T. gondii* infection and immune-driven pregnancy loss (Chen et al., 2024, Chen et al., 2025b). This evidence led us to hypothesize a potential interaction between Trem2 and MTOR proteins. Therefore, we performed molecular docking simulations to model the potential interaction interface between Trem2 and MTOR. A binding energy of approximately -28 kcal/mol was obtained from the docking simulation, indicating a strong binding affinity between Trem2 and MTOR (**Figure 1A**). To validate the physical interaction between Trem2 and MTOR, we first performed co-immunoprecipitation assays. Using an anti-MTOR antibody to immunoprecipitated from Raw264.7 macrophage lysates, we successfully pulled down endogenous Trem2, as detected by Western blotting (**Figure 1B**). Furthermore, to assess their subcellular colocalization, we conducted immunofluorescence staining. Confocal microscopy images revealed a clear overlap of Trem2 (green) and MTOR (red) signals in Raw264.7 macrophages (**Figures 1C, D**). To disrupt the Trem2-MTOR interaction without affecting the structural stability of Trem2 itself, we performed alanine scanning mutagenesis on five key binding residues (GLN-198, ARG-195, LYS-199, SER-171, THR-170) of Trem2, based on the complex model predicted by Pymol molecular docking (**Figure 1E**). Subsequently, we examined the binding ability of mutant to MTOR by co-immunoprecipitation assays (**Figure 1F**). The results showed that these mutants failed to bind to MTOR. Together, these findings demonstrate that Trem2 and MTOR form a specific protein complex in macrophages.

### 3.2 Trem2 deficiency exacerbates MTOR activation in response to *T. gondii* infection

**Figure 2:**
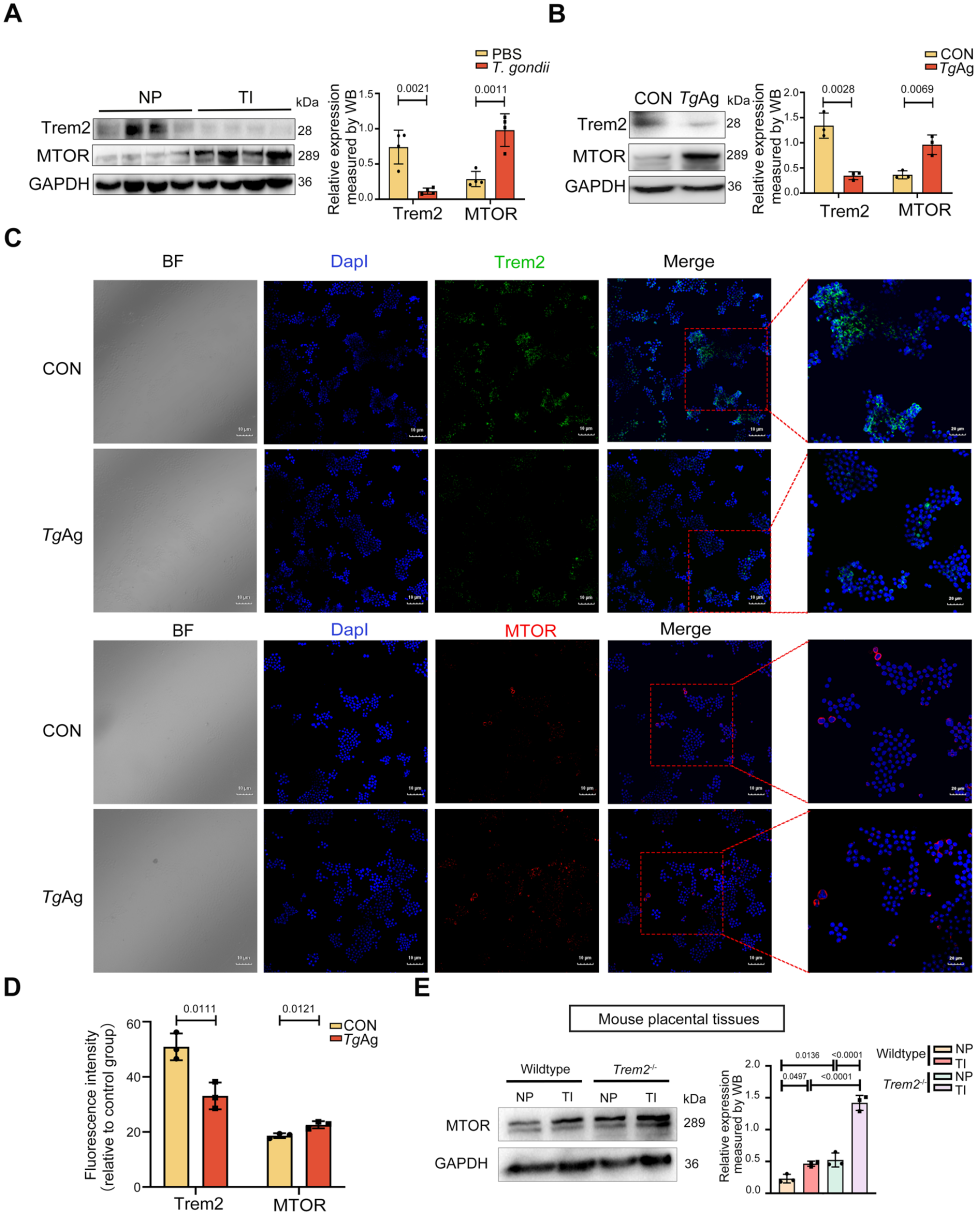
*T. gondii* impairs the Trem2-MTOR interaction in macrophages. **(A)** Trem2 and MTOR protein levels in placentas of wild-type mice under NP or TI at ED17.5 were assessed by Western blot (n = 4). **(B)** Expression of Trem2 and MTOR proteins in Raw264.7 macrophages treated with *Tg*Ag (5 μg/mL, 48h), analyzed by Western blot (n = 3). **(C)** Immunofluorescence was used to visualize the subcellular distribution of Trem2 (green) and MTOR (red) in Raw264.7 macrophages, comparing untreated condition with those stimulated by *Tg*Ag (5 μg/mL, 48h). Cellular nuclei were identified by DAPI staining. **(D)** The fluorescence intensities of Trem2 and MTOR were quantitatively assessed (n = 3). **(E)** Western blot analysis was conducted to determine MTOR expression levels in placentas of wild-type and *Trem2* knockout mice under NP or TI at ED17.5 (n = 4). GAPDH acted as an internal reference. A two-tailed unpaired Student′s *t*-test was applied to A, B, and D, and two-way ANOVA with Sidak′s *post hoc* test was used for **(E)** TI, *T. gondii* infection.

We initially assessed the *in vivo* protein expression of Trem2 and MTOR to explore their functional relationship during *T. gondii* infection. Firstly, by constructing the infected *Trem2^-/-^* pregnancy mouse model, we found that infected *Trem2^-/-^* fetuses exhibited exacerbated intrauterine growth restriction, compared to infected WT controls (**Supplementary Figures 1A-C**). Histological examination of placental tissues by H&E staining also showed more pronounced placental damage in infected *Trem2^-/-^* mice (**Supplementary Figure 1D**). Western blot analysis of placental tissues revealed that Trem2 protein decreased markedly, whereas MTOR showed a marked increase relative to the uninfected group (**Figure 2A**). Given the complexity of the *in vivo* environment, we stimulated Raw264.7 macrophages with *Tg*Ag to simulate *in vitro* infection. We found that this stimulation successfully recapitulated the *in vivo* phenomenon: Trem2 expression decreased, while MTOR expression increased (**Figure 2B**). Immunofluorescence staining further confirmed that after *Tg*Ag stimulation, the fluorescence signal of Trem2 was weakened, while the MTOR signal was enhanced in Raw264.7 macrophages (**Figures 2C, D**). To directly verify the influence of Trem2 on MTOR, we compared Trem2 and MTOR protein expression in placentas of WT versus *Trem2* knockout mouse. Under control conditions, knockout mice exhibited higher basal MTOR expression than their WT counterparts; after *T. gondii* infection, MTOR expression in knockout mice was further increased (**Figure 2E**). This result suggests that the absence of Trem2 predisposes the host to excessive activation of the MTOR pathway during *T. gondii* infection.

Collectively, these data demonstrate that *Trem2* deficiency not only aggravates *T. gondii*-induced fetal growth restriction but leads to excessive MTOR activation.

### 3.3 PKCα functions as a major downstream mediator of the Trem2-MTOR axis upon *T. gondii* infection

**Figure 3:**
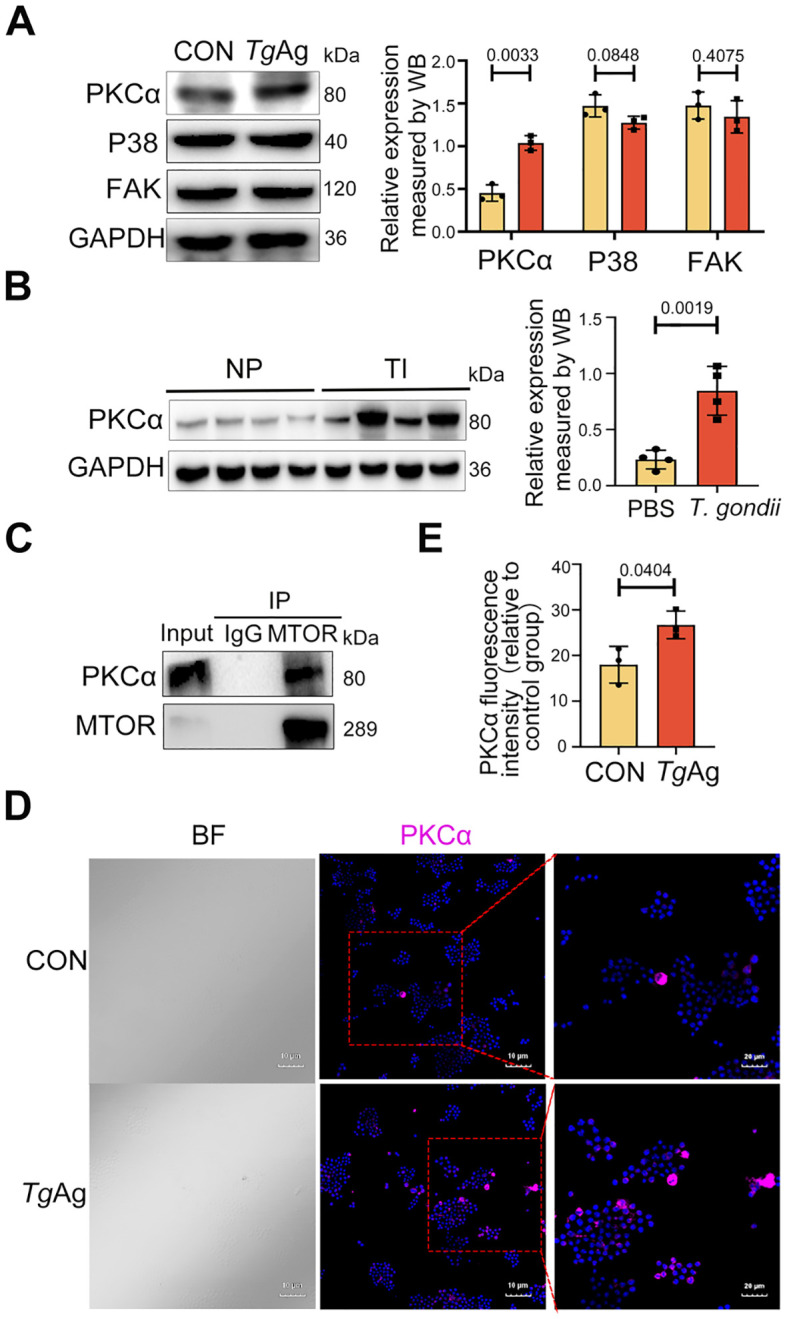
*T. gondii* antigen activates PKCα through the MTOR pathway. **(A)** PKCα, p38, and FAK protein levels in Raw264.7 macrophages following *Tg*Ag exposure (5 μg/mL, 48h) were assessed by Western blot (n = 3). **(B)** Western blot detection of PKCα expression in placentas of wild-type mice at ED17.5 under NP or TI conditions (n = 4). **(C)** Co-immunoprecipitation assay confirming the interaction between PKCα and MTOR. Lysates were immunoprecipitated with anti-MTOR and detected with antibodies against PKCα and MTOR. **(D)** Immunofluorescence visualization of PKCα (magenta) distribution in Raw264.7 cells following *Tg*Ag exposure (5 μg/mL, 48h) stimulated conditions. DAPI was used to stain nuclei. **(E)** The fluorescence intensities of PKCα were quantitatively assessed (n = 3). GAPDH acted as an internal reference. A two-tailed unpaired Student′s *t*-test was applied for statistical analysis. TI, *T. gondii* infection.

After confirming that *Trem2* deficiency leads to excessive MTOR activation upon *T. gondii* infection, then we explored the key downstream effector molecules mediating this pathological effect. PKCα has been identified as a downstream effector of the MTOR pathway in various cellular contexts (Moschetta et al., 2014). Furthermore, PKCα is highly present in macrophages and is known to participate in controlling uterine vascular adaptation during pregnancy (Zhang et al., 2006). In parallel, p38 MAPK was examined as a central mediator of *T. gondii*-induced inflammatory stress, given its documented role in pathogen-triggered cytokine production in infected macrophages (Kim et al., 2005). Focal adhesion kinase (FAK) was also included in our screening panel owing to its established function as a key regulator of MTOR-dependent macrophage migration and adhesion-processes that are essential for decidual macrophage retention and function (Owen et al., 2014). Through candidate molecule screening in *Tg*Ag-stimulated Raw264.7 macrophages, we found that PKCα protein levels were significantly elevated, while other candidate molecules such as P38 and focal adhesion kinase (FAK) showed no significant changes (**Figure 3A**). Based on this, we identified PKCα as a potential downstream effector of the MTOR pathway. To further validate this regulatory relationship, we assessed PKCα expression levels in placentas from mice infected with *T. gondii*, and the results showed a moderate increase in its expression (**Figure 3B**). To establish a direct association between MTOR and PKCα, we further performed co-immunoprecipitation experiments. Using an anti-MTOR antibody, we successfully precipitated PKCα from macrophage lysates, confirming an interaction between the two proteins (**Figure 3C**). Immunofluorescence staining results showed that after *Tg*Ag stimulation, the fluorescence intensity of PKCα (magenta) in macrophages was significantly enhanced, and quantification validated this alteration (**Figures 3D, E**). In summary, this study reveals that PKCα is a key downstream effector of the Trem2-MTOR signaling axis, and *T. gondii* infection participates in host immune regulation by upregulating the MTOR-PKCα cascade signaling.

### 3.4 Trem2 negatively regulates the MTOR-PKCα axis via direct modulation of MTOR expression

**Figure 4:**
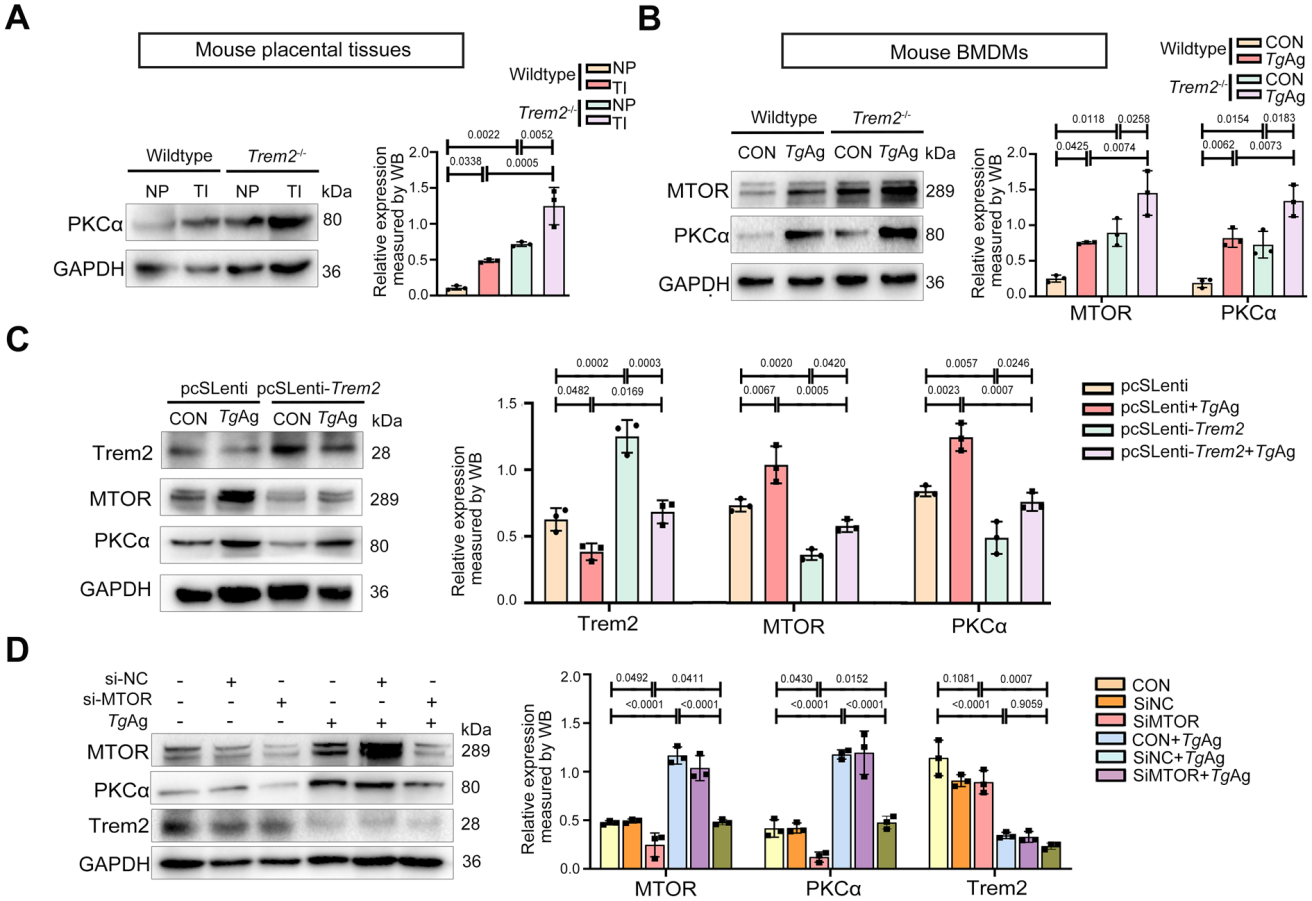
*Trem2* deficiency exacerbates *T. gondii*-induced hyperactivation of the MTOR-PKCα pathway. **(A)** At ED17.5, PKCα protein levels were assessed by Western blot in placentas of wild-type versus *Trem2* knockout mice under NP or TI conditions (n = 3). **(B)** BMDMs isolated from wild-type and *Trem2* knockout mice, were cultured in *Tg*Ag (5 μg/mL, 48h), followed by Western blot detection of MTOR and PKCα protein expression (n = 3). **(C)** Cells were transfected with pcSLenti-empty or pcSLenti-*Trem2*, then treated with *Tg*Ag (5 μg/mL, 48h) as indicated. MTOR and PKCα levels were detected by WB. **(D)** Raw264.7 cells were transfected with si-MTOR (20 nM) or si-NC (20 nM) for 6 h, followed by *Tg*Ag stimulation (5 µg/mL, 48 h). Trem2 levels were then detected by Western blot. GAPDH acted as an internal reference. Two-way ANOVA with Sidak′s *post hoc* test was applied for statistical analysis. TI, *T. gondii* infection.

To comprehensively verify the regulatory effect of Trem2 on the MTOR-PKCα axis, we first examined the expression dynamics of PKCα in placental tissues from wildtype and *Trem2* knockout mice. Following *T. gondii* infection, we observed that the PKCα level in knockout mice was further significantly amplified (**Figure 4A**). To further confirm this phenomenon, we isolated BMDMs from WT mouse and performed Western blot analysis after *Tg*Ag stimulation. MTOR and PKCα protein levels were markedly higher in *Trem2* knockout BMDMs than in WT cells, and this difference became even more pronounced upon *Tg*Ag stimulation (**Figure 4B**). These results indicate that *Trem2* deficiency promotes the activation of the MTOR- PKCα axis, and this effect is further exacerbated under inflammatory stimulation. To reversely validate the negative regulatory function of Trem2, we transfected Raw264.7 macrophages with *Trem2*-*DAP12* overexpression lentiviral vector. Compared with the empty vector control, overexpression of *Trem2* significantly inhibited the protein expression of MTOR and PKCα; while after *Tg*Ag stimulation, the levels of both in overexpressing cells showed a rebound trend (**Figure 4C**). These findings suggest that Trem2 directly negatively regulates the MTOR-PKCα axis, an inhibition that can be partially reversed by *T. gondii* infection. To ultimately determine whether MTOR is a necessary mediator for PKCα regulation downstream of Trem2, we performed siRNA-mediated *MTOR* knockdown experiments in macrophages. The results showed that after efficient silencing of MTOR expression, PKCα protein levels were significantly reduced to near baseline levels, while Trem2 itself showed no significant change after *MTOR* knockdown (**Figure 4D**). In summary, this study confirms that Trem2 affects PKCα levels by negatively regulating MTOR expression; *Trem2* deficiency leads to excessive activation of the MTOR-PKCα axis.

## 4 Discussion

*T. gondii*, one of the most prevalent and well-adapted intracellular parasites, is a significant infectious factor leading to APC (He et al., 2025). The successful progression of pregnancy depends on a finely tuned immune equilibrium at the maternal-fetal interface, which facilitates embryo implantation, fetal growth, and delivery (Levenson et al., 2025; Yang et al., 2025). Macrophages lie at the heart of this immune regulation, as they are essential for preserving immune homeostasis and promoting fetal development (Yang et al., 2025). In pregnant women, infection with pathogenic strains such as RH can lead to recurrent pregnancy loss (RPL) through various mechanisms that induce macrophage dysfunction, including the downregulation of adhesion molecules (such as E-cadherin) and disruption of the balance of cell proliferation markers (abnormal Ki67 expression and elevated levels of Bcl-2 and P27kip) (Yuan et al., 2025). Studies have shown that macrophages highly express Trem2 to promote lipid catabolism and suppress inflammation (Zhu et al., 2024). These findings suggest that Trem2 is abundantly present on macrophages and serves two functions: modulating metabolism and controlling inflammatory responses. The molecular mechanism underlying *T. gondii*-induced Trem2 downregulation has been partially elucidated. Our previous study demonstrated that *T. gondii* infection activates the transcription factor activating transcription factor 3 (ATF3) in host macrophages, which directly binds to the promoter region of the Trem2 gene and represses its transcription, leading to decreased Trem2 expression (Geng et al., 2025). Notably, this downregulation does not appear to be an isolated event. *T. gondii* has been shown to simultaneously target multiple immunoregulatory receptors on decidual macrophages. For example, *T. gondii* downregulates the inhibitory receptor LILRB4, thereby promoting M1 polarization of decidual macrophages and APC (Li et al., 2017). Likewise, Tim-3 expression is also reduced following infection, accompanied by decreased expression of M2-associated molecules (CD163, CD206 and CD209) and enhanced inflammatory activation (Zhang et al., 2019). Collectively, these findings suggest that coordinated suppression of immunoregulatory receptors may represent a common strategy by which *T. gondii* disrupts decidual macrophage homeostasis. Whether Trem2 and these related receptors share common upstream regulatory mechanisms remains to be determined. Furthermore, while Trem2 has been implicated in host defense against other intracellular parasites, for instance, it governs Kupffer cell activation and resistance to *Plasmodium berghei* liver stage infection, it is unclear whether pathogen-induced Trem2 downregulation is a conserved immune-evasion strategy across different intracellular or placenta-infecting parasites (Gonçalves et al., 2013). Comparative studies across diverse parasite species will be valuable to address this question and may reveal broadly targetable nodes in the host-pathogen interaction network.

Trem2 is an immunoregulatory receptor expressed on the surface of myeloid cells (Li et al., 2025). Acting as a signaling hub, it is essential for mediating immune responses (Yeh et al., 2017). It not only regulates the phagocytic function of macrophages but also modulates the intensity of inflammatory responses and promotes a switch from the pro-inflammatory M1 phenotype toward the anti-inflammatory M2 phenotype (Peng et al., 2025b). Recently, upregulation of Trem2 was shown to promote microglial polarization toward the M2 anti-inflammatory phenotype and increase BDNF secretion via PI3K/Akt signaling activation. This led to enhanced hippocampal neurogenesis and better spatial cognition in APP/PS1 mice (Peng et al., 2025a). To elucidate the downstream signaling mechanism of Trem2 in macrophages, we employed bioinformatics prediction combined with co-immunoprecipitation validation and, for the first time, discovered a high-affinity interaction between Trem2 and MTOR, and further validated the key amino acid residues mediating this binding. As a key kinase that integrates cellular metabolism, autophagy, and inflammatory responses, MTOR plays a central role in regulating macrophage function (Zhou et al., 2025). Several studies have suggested a link between Trem2 and autophagy. For instance, in a *Trem2*-deficient mouse model, Uland et al. observed an increased number of autophagic vesicles due to impaired MTOR signaling (Ou-Yang et al., 2023; Lior et al., 2025). However, in the context of *T. gondii* infection in the present study, *Trem2* deficiency led to significant upregulation and hyperactivation of MTOR. Furthermore, studies have observed elevated IFNγ levels in *T. gondii*-infected 4E KI mice (Leroux et al., 2020). This could be explained by a higher parasite load together with genetic susceptibility, which worsens inflammation in these knockout mice, thereby driving macrophages toward a pathological pro-inflammatory phenotype and ultimately exacerbating inflammatory damage and APC at the maternal-fetal interface.

In the physiological and pathological processes of the placenta, MTOR signaling plays a particularly critical role: its dysregulation is closely associated with various APC (Wang et al., 2009). Studies have shown that in human fetal growth restriction and its animal models, placental MTOR signaling is significantly suppressed; conversely, in complications involving excessive fetal growth, placental MTOR activity is abnormally elevated (Kramer et al., 2023). In the present study, we detected a marked increase in MTOR protein levels in placentas following *T. gondii* infection. This finding aligns with previous reports: *T. gondii* infection enhances host MTOR signaling and MTOR-dependent mRNA translation, a mechanism believed to favor host cell survival and parasite replication (Leroux et al., 2018; Martin et al., 2024). Further supporting the notion that MTOR is a key target of parasite survival strategies, we found that inhibiting MTOR expression using small interfering RNA significantly attenuated the damaging effects induced by *T. gondii* infection. However, given its essential role in placental development, systemic inhibition of MTOR during pregnancy carries substantial risks. Therefore, potential therapeutic approaches should avoid global MTOR suppression and instead focus on cell-type-specific targeting, particularly toward placental macrophages. Alternatively, restoring or stabilizing the Trem2-MTOR interaction may represent a more selective approach to correcting infection-induced signaling imbalance while preserving physiological MTOR function. Previous research on MTOR signaling has largely focused on its upstream regulatory networks, such as how the PI3K/AKT pathway responds to growth factors and nutrients in the microenvironment (Xu et al., 2026). However, the downstream effector molecules of MTOR in the context of *T. gondii* infection remain unclear. To address this gap, we systematically screened multiple candidate molecules, including P38 and FAK, and found that only PKCα expression was significantly altered following infection, with a trend highly consistent with that of MTOR. This suggests that PKCα may be a key downstream effector molecule of MTOR, serving as a critical signal transducer downstream of the Trem2-MTOR axis.

PKCα belongs to the conventional PKC family and functions as a serine/threonine kinase that participates broadly in signal transduction governing cell proliferation, differentiation, apoptosis, and inflammatory responses (Zhu et al., 2025a). Recent studies have confirmed that *T. gondii* infection triggers conventional PKC isoforms (including PKCα and PKCβ) to become activated and translocate to the plasma membrane, while macrophages deficient in PKCβ exhibit impaired MAPK activation, suggesting that PKC family members perform indispensable signal transduction functions (Masek et al., 2006). In the present study, we observed a significant upregulation of PKCα protein levels in both macrophages and placental tissues following *T. gondii* infection. Co-immunoprecipitation assays further confirmed the interaction between PKCα and MTOR. More importantly, appropriate activity of PKCα signaling is crucial for maintaining homeostasis at the maternal-fetal interface. In trophoblast cells, PKCα promotes the migration and invasion of extravillous trophoblasts by receiving regulatory signals from syndecan-4, establishing it as a key signaling molecule for proper placental formation (Jeyarajah et al., 2019). However, in the context of *Trem2* deficiency in macrophages in this study, the upregulation of PKCα was further amplified, while *Trem2* overexpression partially suppressed this effect, which was accompanied by APC. Based on these findings, we propose that PKCα serves as a key downstream mediator of Trem2-MTOR axis. In summary, this study reveals for the first time the central regulatory role of the Trem2-MTOR-PKCα signaling axis in APC induced by *T. gondii* infection.

### Limitations of the study

First, the relevance and conservation of Trem2-MTOR-PKCα pathway in human placental tissues remains unclear, as the present study was conducted in murine models and macrophage systems. Second, future studies should aim to resolve the structural basis of the Trem2-MTOR signaling complex using cryo-electron microscopy (cryo-EM), which is well suited for membrane-associated signaling complexes because it preserves near-native conformations without requiring crystallization (Chen et al., 2025a; Chien et al., 2025). Nevertheless, challenges including membrane protein purification, conformational heterogeneity and complex stability remain to be addressed. Complementary approaches, such as hydrogen-deuterium exchange mass spectrometry (HDX-MS) and chemical cross-linking mass spectrometry (XL-MS), may help capture infection-induced conformational dynamics and protein-protein interaction and interaction interfaces that are not readily accessible by cryo-EM alone (Castel et al., 2025; Zou et al., 2026). Additionally, future validation should be extended to clinically relevant *T. gondii* strains [type I (RH), type II (ME49), and type III strains] as well as primary human decidual macrophages and trophoblast-derived cells, to better define species- and strain-specific effects.

## Conclusion

Since Trem2 negatively regulates the MTOR-PKCα axis through direct interaction and *T. gondii* infection disrupts this protective mechanism to drive adverse pregnancy outcomes, the Trem2-MTOR-PKCα axis represents a promising therapeutic target for congenital toxoplasmosis.

## Data Availability

The datasets presented in this study can be found in online repositories. The names of the repository/repositories and accession number(s) can be found in the article/**Supplementary Material**.
