# Supplementary material for: Trem2 negatively regulates the MTOR-PKCα axis to protect against Toxoplasma gondii-induced adverse pregnancy outcomes
Source: Front Cell Infect Microbiol. 2026 Jul 30;16:1897580. doi: 10.3389/fcimb.2026.1897580 (PMC13468792; doi:10.3389/fcimb.2026.1897580)
Supplement: Supplementary file 1 [file DataSheet1.docx]

**supplementary Figures**

**Fig. S1.**

*Trem2* deficiency exacerbates placental hemorrhage and necrosis upon *T. gondii* infection in mice. (A) Representative images of fetuses from wild-type and *Trem2*^–/–^ mice under NP or TI conditions. (B and C) Fetal development was assessed by crown-rump length (CRL), occipito-frontal diameter (OFD), and fetal weight. Each data point represents an individual fetus (n = 6 dams per group). (D) Representative H&E-stained placental sections from wild-type and *Trem2*^–/–^ mice under NP or TI conditions. Placentas from the TI group displayed extensive hemorrhagic necrosis (red arrows) across all placental compartments, including the labyrinth zone (LZ), decidua (De), and junctional zone (JZ). Statistical analyses were performed using a two‑tailed unpaired Student’s *t*‑test.
