# Supplementary material for: Trem2 negatively regulates the MTOR-PKCα axis to protect against Toxoplasma gondii-induced adverse pregnancy outcomes
Source: Front Cell Infect Microbiol. 2026 Jul 30;16:1897580. doi: 10.3389/fcimb.2026.1897580 (PMC13468792; doi:10.3389/fcimb.2026.1897580)
Supplement: Supplementary file 2 [file Table1.docx]

The whole uncropped images of the original western blots for all replicates that contributed to the quantitative analysis were shown as followed (Table 1).

Table 1. **Involved figures and number**

|  | **Involved figures** | **Number of original western blots** |
| --- | --- | --- |
| **1** | Figure 1 B | **3** (Trem2, MTOR) |
| **2** | Figure 1 F | **3** (Trem2, MTOR) |
| **3** | Figure 2 A | **4** (Trem2, MTOR and GAPDH) |
| **4** | Figure 2 B | **6** (Trem2 and GAPDH); (MTOR and GAPDH) |
| **5** | Figure 2 E | **9** (MTOR and GAPDH) |
| **6** | Figure 3 A | **8** (MTOR and GAPDH); (PKCα and GAPDH); (P38, FAK and GAPDH) |
| **7** | Figure 3 B | **3** (PKCα and GAPDH) |
| **8** | Figure 3 C | **3** (MTOR, PKCα and GAPDH) |
| **9** | Figure 4 A | **9** (PKCα and GAPDH) |
| **10** | Figure 4 B | **18** (MTOR and GAPDH); (PKCα and GAPDH) |
| **11** | Figure 4 C | **24** (Trem2 and GAPDH); (MTOR and GAPDH); (PKCα and GAPDH) |
| **12** | Figure 4 D | **24** (MTOR and GAPDH); (PKCα and GAPDH); (Trem2 and GAPDH); |

**1.Figure 1B** **Trem2 expression detected following co‑IP using an anti‑MTOR antibody.**

**(1) Experiment 1**

Marker A: Precision Plus Protein Dual Color Standards (10-250kDa), Catalog #1610374;

1: input; 2: IgG; 3:IP;

Marker A: Precision Plus Protein Dual Color Standards (10-250kDa), Catalog #1610374;

1: input; 2: IgG; 3:IP;

**Merged figure**

**1.Figure 1F Trem2 mutant binding to MTOR assessed by co‑immunoprecipitation following alanine‑scanning mutagenesis.**

**(1) Experiment 1**

Marker A: Precision Plus Protein Dual Color Standards (10-250kDa), Catalog #1610374;

1: input (WT Trem2 + MTOR); 2: IgG control (WT); 3: IP: anti-MTOR (WT); 4: input (Trem2 mutant + MTOR); 5: IgG control (mutant); 6: IP: anti-MTOR (mutant);

Marker A: Precision Plus Protein Dual Color Standards (10-250kDa), Catalog #1610374;

1: input (WT Trem2 + MTOR); 2: IgG control (WT); 3: IP: anti-MTOR (WT); 4: input (Trem2 mutant + MTOR); 5: IgG control (mutant); 6: IP: anti-MTOR (mutant);

**Merged figure**

**2.Figure 2A Expression levels of Trem2 and MTOR following exposure to *T.*** ***gondii*.**

**(1) Experiment 1**

Marker A: Precision Plus Protein Dual Color Standards (10-250kDa), Catalog #1610374;

1-4: PBS; 5-8: *T. gondii*;

**(2) Experiment 2**

Marker A: Precision Plus Protein Dual Color Standards (10-250kDa), Catalog #1610374;

1-4: PBS; 5-8: *T. gondii*;

Marker A: Precision Plus Protein Dual Color Standards (10-250kDa), Catalog #1610374;

1-4: PBS; 5-8: *T. gondii*;

**Merged figure**

**2.Figure 2B Expression levels of Trem2 and MTOR following exposure to *T. gondii* antigens.**

**(1) Experiment 1**

Marker A: Precision Plus Protein Dual Color Standards (10-250kDa), Catalog #1610374;

1-4: Con; 5-8: *Tg*Ag;

*Tg*Ag: *T. gondii* antigens

Marker A: Precision Plus Protein Dual Color Standards (10-250kDa), Catalog #1610374;

1-4: Con; 5-8: *Tg*Ag;

*Tg*Ag: *T. gondii* antigens

**Merged figure**

**(2) Experiment 2**

Marker A: Precision Plus Protein Dual Color Standards (10-250kDa), Catalog #1610374;

1-3: Con; 4-6: *Tg*Ag;

*Tg*Ag: *T. gondii* antigens

Marker A: Precision Plus Protein Dual Color Standards (10-250kDa), Catalog #1610374;

1-3: Con; 4-6: *Tg*Ag;

*Tg*Ag: *T. gondii* antigens

**Merged figure**

**2.Figure 2E MTOR expression in placentas of WT and *Trem2* KO mice under NP or TI conditions.**

**(1) Experiment 1**

Marker A: Precision Plus Protein Dual Color Standards (10-250kDa), Catalog #1610374;

1: wildtype NP; 2: wildtype TI; 3: *Trem*2^-/-^ NP; 4: *Trem*2^-/-^ TI;

Marker A: Precision Plus Protein Dual Color Standards (10-250kDa), Catalog #1610374;

1: wildtype NP; 2: wildtype TI; 3: *Trem*2^-/-^ NP; 4: *Trem*2^-/-^ TI;

**Merged figure**

**(1) Experiment 1**

Marker A: Precision Plus Protein Dual Color Standards (10-250kDa), Catalog #1610374;

1: wildtype NP; 2: wildtype TI; 3: *Trem*2^-/-^ NP; 4: *Trem*2^-/-^ TI;

Marker A: Precision Plus Protein Dual Color Standards (10-250kDa), Catalog #1610374;

1: wildtype NP; 2: wildtype TI; 3: *Trem*2^-/-^ NP; 4: *Trem*2^-/-^ TI;

**Merged figure**

**(1) Experiment 1**

Marker A: Precision Plus Protein Dual Color Standards (10-250kDa), Catalog #1610374;

1: wildtype NP; 2: wildtype TI; 3: *Trem*2^-/-^ NP; 4: *Trem*2^-/-^ TI;

Marker A: Precision Plus Protein Dual Color Standards (10-250kDa), Catalog #1610374;

1: wildtype NP; 2: wildtype TI; 3: *Trem*2^-/-^ NP; 4: *Trem*2^-/-^ TI;

**Merged figure**

**3.Figure 3A Expression levels of PKCα following exposure to *T. gondii* antigens.**

**(1) Experiment 1**

Marker A: Precision Plus Protein Dual Color Standards (10-250kDa), Catalog #1610374;

1-4: Con; 5-8: *Tg*Ag;

*Tg*Ag: *T. gondii* antigens

Marker A: Precision Plus Protein Dual Color Standards (10-250kDa), Catalog #1610374;

1-4: Con; 5-8: *Tg*Ag;

*Tg*Ag: *T. gondii* antigens

**Merged figure**

**(2) Experiment 2**

Marker A: Precision Plus Protein Dual Color Standards (10-250kDa), Catalog #1610374;

1: Con; 2: *Tg*Ag; 3: Con; 4: *Tg*Ag; 5: Con; 6: *Tg*Ag;

*Tg*Ag: *T. gondii* antigens

Marker A: Precision Plus Protein Dual Color Standards (10-250kDa), Catalog #1610374;

1: Con; 2: *Tg*Ag; 3: Con; 4: *Tg*Ag; 5: Con; 6: *Tg*Ag;

*Tg*Ag: *T. gondii* antigens

**(3) Experiment 3**

Marker A: Precision Plus Protein Dual Color Standards (10-250kDa), Catalog #1610374;

1: Con; 2: *Tg*Ag; 3: Con; 4: *Tg*Ag; 5: Con; 6: *Tg*Ag;

*Tg*Ag: *T. gondii* antigens

Marker A: Precision Plus Protein Dual Color Standards (10-250kDa), Catalog #1610374;

1: Con; 2: *Tg*Ag; 3: Con; 4: *Tg*Ag; 5: Con; 6: *Tg*Ag;

*Tg*Ag: *T. gondii* antigens

**Merged figure**

**3.Figure 3B PKCα expression in placentas of wild-type mice under NP or TI conditions.**

Marker A: Precision Plus Protein Dual Color Standards (10-250kDa), Catalog #1610374;

1-4: PBS; 5-8: *T. gondii*;

Marker A: Precision Plus Protein Dual Color Standards (10-250kDa), Catalog #1610374;

1-4: PBS; 5-8: *T. gondii*;

**Merged figure**

**3.Figure 3C PKCα expression detected following co‑IP using an anti‑MTOR antibody.**

Marker A: Precision Plus Protein Dual Color Standards (10-250kDa), Catalog #1610374;

1: input; 2: IgG; 3:IP; 4: input; 5: IgG; 6:IP;

Marker A: Precision Plus Protein Dual Color Standards (10-250kDa), Catalog #1610374;

1: input; 2: IgG; 3:IP; 4: input; 5: IgG; 6:IP;

**Merged figure**

**3.Figure 4A PKCα expression in placentas of wild-type and Trem2 knockout mice under NP or TI conditions**

**(1) Experiment 1**

Marker B: Blue Plus® Protein Marker (14-100kDa), Catalog DM101-01;

1: wildtype NP; 2: wildtype TI; 3: *Trem*2^-/-^ NP; 4: *Trem*2^-/-^ TI;

Marker B: Blue Plus® Protein Marker (14-100kDa), Catalog DM101-01;

1: wildtype NP; 2: wildtype TI; 3: *Trem*2^-/-^ NP; 4: *Trem*2^-/-^ TI;

**Merged figure**

**(1) Experiment 1**

Marker B: Blue Plus® Protein Marker (14-100kDa), Catalog DM101-01;

1: wildtype NP; 2: wildtype TI; 3: *Trem*2^-/-^ NP; 4: *Trem*2^-/-^ TI;

Marker B: Blue Plus® Protein Marker (14-100kDa), Catalog DM101-01;

1: wildtype NP; 2: wildtype TI; 3: *Trem*2^-/-^ NP; 4: *Trem*2^-/-^ TI;

**Merged figure**

**(1) Experiment 1**

Marker B: Blue Plus® Protein Marker (14-100kDa), Catalog DM101-01;

1: wildtype NP; 2: wildtype TI; 3: *Trem*2^-/-^ NP; 4: *Trem*2^-/-^ TI;

Marker B: Blue Plus® Protein Marker (14-100kDa), Catalog DM101-01;

1: wildtype NP; 2: wildtype TI; 3: *Trem*2^-/-^ NP; 4: *Trem*2^-/-^ TI;

**Merged figure**

**3.Figure 4B MTOR and PKCα expression in BMDMs isolated from wild-type and *Trem2* knockout mice following *Tg*Ag stimulation.**

**(1) Experiment 1**

Marker A: Precision Plus Protein Dual Color Standards (10-250kDa), Catalog #1610374;

1: wildtype CON; 2: wildtype + *Tg*Ag; 3: *Trem*2^-/-^ CON; 4: *Trem*2^-/-^ + *Tg*Ag;

Marker A: Precision Plus Protein Dual Color Standards (10-250kDa), Catalog #1610374;

1: wildtype CON; 2: wildtype + *Tg*Ag; 3: *Trem*2^-/-^ CON; 4: *Trem*2^-/-^ + *Tg*Ag;

**Merged figure**

**(1) Experiment 1**

Marker A: Precision Plus Protein Dual Color Standards (10-250kDa), Catalog #1610374;

1: wildtype CON; 2: wildtype + *Tg*Ag; 3: *Trem*2^-/-^ CON; 4: *Trem*2^-/-^ + *Tg*Ag;

Marker A: Precision Plus Protein Dual Color Standards (10-250kDa), Catalog #1610374;

1: wildtype CON; 2: wildtype + *Tg*Ag; 3: *Trem*2^-/-^ CON; 4: *Trem*2^-/-^ + *Tg*Ag;

**Merged figure**

**(1) Experiment 1**

Marker A: Precision Plus Protein Dual Color Standards (10-250kDa), Catalog #1610374;

1: wildtype CON; 2: wildtype + *Tg*Ag; 3: *Trem*2^-/-^ CON; 4: *Trem*2^-/-^ + *Tg*Ag;

Marker A: Precision Plus Protein Dual Color Standards (10-250kDa), Catalog #1610374;

1: wildtype CON; 2: wildtype + *Tg*Ag; 3: *Trem*2^-/-^ CON; 4: *Trem*2^-/-^ + *Tg*Ag;

**Merged figure**

**(2) Experiment 2**

Marker A: Precision Plus Protein Dual Color Standards (10-250kDa), Catalog #1610374;

1: wildtype CON; 2: wildtype + *Tg*Ag; 3: *Trem*2^-/-^ CON; 4: *Trem*2^-/-^ + *Tg*Ag;

Marker A: Precision Plus Protein Dual Color Standards (10-250kDa), Catalog #1610374;

1: wildtype CON; 2: wildtype + *Tg*Ag; 3: *Trem*2^-/-^ CON; 4: *Trem*2^-/-^ + *Tg*Ag;

**Merged figure**

**(2) Experiment 2**

Marker A: Precision Plus Protein Dual Color Standards (10-250kDa), Catalog #1610374;

1: wildtype CON; 2: wildtype + *Tg*Ag; 3: *Trem*2^-/-^ CON; 4: *Trem*2^-/-^ + *Tg*Ag;

Marker A: Precision Plus Protein Dual Color Standards (10-250kDa), Catalog #1610374;

1: wildtype CON; 2: wildtype + *Tg*Ag; 3: *Trem*2^-/-^ CON; 4: *Trem*2^-/-^ + *Tg*Ag;

**Merged figure**

**(2) Experiment 2**

Marker A: Precision Plus Protein Dual Color Standards (10-250kDa), Catalog #1610374;

1: wildtype CON; 2: wildtype + *Tg*Ag; 3: *Trem*2^-/-^ CON; 4: *Trem*2^-/-^ + *Tg*Ag;

Marker A: Precision Plus Protein Dual Color Standards (10-250kDa), Catalog #1610374;

1: wildtype CON; 2: wildtype + *Tg*Ag; 3: *Trem*2^-/-^ CON; 4: *Trem*2^-/-^ + *Tg*Ag;

**Merged figure**

**3.Figure 4C MTOR and PKCα expression in Raw264.7 macrophages transfected with empty vector or *Trem2* overexpression construct, with or without *Tg*Ag stimulation.**

**(1) Experiment 1**

Marker A: Precision Plus Protein Dual Color Standards (10-250kDa), Catalog #1610374;

1: Vector + Control; 2: Vector + *Tg*Ag; 3: *Trem2* OE + Control; 4: *Trem2* OE + *Tg*Ag;

Marker A: Precision Plus Protein Dual Color Standards (10-250kDa), Catalog #1610374;

1: Vector + Control; 2: Vector + *Tg*Ag; 3: *Trem2* OE + Control; 4: *Trem2* OE + *Tg*Ag;

**(1) Experiment 1**

Marker A: Precision Plus Protein Dual Color Standards (10-250kDa), Catalog #1610374;

1: Vector + Control; 2: Vector + *Tg*Ag; 3: *Trem2* OE + Control; 4: *Trem2* OE + *Tg*Ag;

Marker A: Precision Plus Protein Dual Color Standards (10-250kDa), Catalog #1610374;

1: Vector + Control; 2: Vector + *Tg*Ag; 3: *Trem2* OE + Control; 4: *Trem2* OE + *Tg*Ag;

**(1) Experiment 1**

Marker A: Precision Plus Protein Dual Color Standards (10-250kDa), Catalog #1610374;

1: Vector + Control; 2: Vector + *Tg*Ag; 3: *Trem2* OE + Control; 4: *Trem2* OE + *Tg*Ag;

Marker A: Precision Plus Protein Dual Color Standards (10-250kDa), Catalog #1610374;

1: Vector + Control; 2: Vector + *Tg*Ag; 3: *Trem2* OE + Control; 4: *Trem2* OE + *Tg*Ag;

**(2) Experiment 2**

Marker A: Precision Plus Protein Dual Color Standards (10-250kDa), Catalog #1610374;

1: Vector + Control; 2: Vector + *Tg*Ag; 3: *Trem2* OE + Control; 4: *Trem2* OE + *Tg*Ag;

Marker A: Precision Plus Protein Dual Color Standards (10-250kDa), Catalog #1610374;

1: Vector + Control; 2: Vector + *Tg*Ag; 3: *Trem2* OE + Control; 4: *Trem2* OE + *Tg*Ag;

**Merged figure**

**(2) Experiment 2**

Marker A: Precision Plus Protein Dual Color Standards (10-250kDa), Catalog #1610374;

1: Vector + Control; 2: Vector + *Tg*Ag; 3: *Trem2* OE + Control; 4: *Trem2* OE + *Tg*Ag;

Marker A: Precision Plus Protein Dual Color Standards (10-250kDa), Catalog #1610374;

1: Vector + Control; 2: Vector + *Tg*Ag; 3: *Trem2* OE + Control; 4: *Trem2* OE + *Tg*Ag;

**Merged figure**

**(2) Experiment 2**

Marker A: Precision Plus Protein Dual Color Standards (10-250kDa), Catalog #1610374;

1: Vector + Control; 2: Vector + *Tg*Ag; 3: *Trem2* OE + Control; 4: *Trem2* OE + *Tg*Ag;

Marker A: Precision Plus Protein Dual Color Standards (10-250kDa), Catalog #1610374;

1: Vector + Control; 2: Vector + *Tg*Ag; 3: *Trem2* OE + Control; 4: *Trem2* OE + *Tg*Ag;

**Merged figure**

**(3) Experiment 3**

Marker A: Precision Plus Protein Dual Color Standards (10-250kDa), Catalog #1610374;

1: Vector + Control; 2: Vector + *Tg*Ag; 3: *Trem2* OE + Control; 4: *Trem2* OE + *Tg*Ag;

Marker A: Precision Plus Protein Dual Color Standards (10-250kDa), Catalog #1610374;

1: Vector + Control; 2: Vector + *Tg*Ag; 3: *Trem2* OE + Control; 4: *Trem2* OE + *Tg*Ag;

**Merged figure**

**(3) Experiment 3**

Marker A: Precision Plus Protein Dual Color Standards (10-250kDa), Catalog #1610374;

1: Vector + Control; 2: Vector + *Tg*Ag; 3: *Trem2* OE + Control; 4: *Trem2* OE + *Tg*Ag;

Marker A: Precision Plus Protein Dual Color Standards (10-250kDa), Catalog #1610374;

1: Vector + Control; 2: Vector + *Tg*Ag; 3: *Trem2* OE + Control; 4: *Trem2* OE + *Tg*Ag;

**Merged figure**

**(3) Experiment 3**

Marker A: Precision Plus Protein Dual Color Standards (10-250kDa), Catalog #1610374;

1: Vector + Control; 2: Vector + *Tg*Ag; 3: *Trem2* OE + Control; 4: *Trem2* OE + *Tg*Ag;

Marker A: Precision Plus Protein Dual Color Standards (10-250kDa), Catalog #1610374;

1: Vector + Control; 2: Vector + *Tg*Ag; 3: *Trem2* OE + Control; 4: *Trem2* OE + *Tg*Ag;

**Merged figure**

**3.Figure 4D Trem2 and MTOR expression in Raw264.7 macrophages transfected with si-NC or si-MTOR, with or without *Tg*Ag stimulation.**

**(1) Experiment 1**

Marker A: Precision Plus Protein Dual Color Standards (10-250kDa), Catalog #1610374;

1: Control; 2: si-NC; 3: si-MTOR; 4: Control + *Tg*Ag; 5: si-NC + *Tg*Ag; 6: si-MTOR + *Tg*Ag;

Marker A: Precision Plus Protein Dual Color Standards (10-250kDa), Catalog #1610374;

1: Control; 2: si-NC; 3: si-MTOR; 4: Control + *Tg*Ag; 5: si-NC + *Tg*Ag; 6: si-MTOR + *Tg*Ag;

**Merged figure**

**(1) Experiment 1**

Marker A: Precision Plus Protein Dual Color Standards (10-250kDa), Catalog #1610374;

1: Control; 2: si-NC; 3: si-MTOR; 4: Control + *Tg*Ag; 5: si-NC + *Tg*Ag; 6: si-MTOR + *Tg*Ag;

Marker A: Precision Plus Protein Dual Color Standards (10-250kDa), Catalog #1610374;

1: Control; 2: si-NC; 3: si-MTOR; 4: Control + *Tg*Ag; 5: si-NC + *Tg*Ag; 6: si-MTOR + *Tg*Ag;

**Merged figure**

**(1) Experiment 1**

Marker A: Precision Plus Protein Dual Color Standards (10-250kDa), Catalog #1610374;

1: Control; 2: si-NC; 3: si-MTOR; 4: Control + *Tg*Ag; 5: si-NC + *Tg*Ag; 6: si-MTOR + *Tg*Ag;

Marker A: Precision Plus Protein Dual Color Standards (10-250kDa), Catalog #1610374;

1: Control; 2: si-NC; 3: si-MTOR; 4: Control + *Tg*Ag; 5: si-NC + *Tg*Ag; 6: si-MTOR + *Tg*Ag;

**Merged figure**

**(2) Experiment 2**

Marker A: Precision Plus Protein Dual Color Standards (10-250kDa), Catalog #1610374;

1: Control; 2: si-NC; 3: si-MTOR; 4: Control + *Tg*Ag;5: si-NC + *Tg*Ag; 6: si-MTOR + *Tg*Ag;

Marker A: Precision Plus Protein Dual Color Standards (10-250kDa), Catalog #1610374;

1: Control; 2: si-NC; 3: si-MTOR; 4: Control + *Tg*Ag;5: si-NC + *Tg*Ag; 6: si-MTOR + *Tg*Ag;

**Merged figure**

**(2) Experiment 2**

Marker A: Precision Plus Protein Dual Color Standards (10-250kDa), Catalog #1610374;

1: Control; 2: si-NC; 3: si-MTOR; 4: Control + *Tg*Ag;5: si-NC + *Tg*Ag; 6: si-MTOR + *Tg*Ag;

Marker A: Precision Plus Protein Dual Color Standards (10-250kDa), Catalog #1610374;

1: Control; 2: si-NC; 3: si-MTOR; 4: Control + *Tg*Ag;5: si-NC + *Tg*Ag; 6: si-MTOR + *Tg*Ag;

**Merged figure**

**(2) Experiment 2**

Marker A: Precision Plus Protein Dual Color Standards (10-250kDa), Catalog #1610374;

1: Control; 2: si-NC; 3: si-MTOR; 4: Control + *Tg*Ag;5: si-NC + *Tg*Ag; 6: si-MTOR + *Tg*Ag;

Marker A: Precision Plus Protein Dual Color Standards (10-250kDa), Catalog #1610374;

1: Control; 2: si-NC; 3: si-MTOR; 4: Control + *Tg*Ag;5: si-NC + *Tg*Ag; 6: si-MTOR + *Tg*Ag;

**Merged figure**

**(3) Experiment 3**

Marker A: Precision Plus Protein Dual Color Standards (10-250kDa), Catalog #1610374;

1: Control; 2: si-NC; 3: si-MTOR; 4: Control + *Tg*Ag;5: si-NC + *Tg*Ag; 6: si-MTOR + *Tg*Ag;

Marker A: Precision Plus Protein Dual Color Standards (10-250kDa), Catalog #1610374;

1: Control; 2: si-NC; 3: si-MTOR; 4: Control + *Tg*Ag;5: si-NC + *Tg*Ag; 6: si-MTOR + *Tg*Ag;

**(3) Experiment 3**

Marker A: Precision Plus Protein Dual Color Standards (10-250kDa), Catalog #1610374;

1: Control; 2: si-NC; 3: si-MTOR; 4: Control + *Tg*Ag;5: si-NC + *Tg*Ag; 6: si-MTOR + *Tg*Ag;

Marker A: Precision Plus Protein Dual Color Standards (10-250kDa), Catalog #1610374;

1: Control; 2: si-NC; 3: si-MTOR; 4: Control + *Tg*Ag;5: si-NC + *Tg*Ag; 6: si-MTOR + *Tg*Ag;

**(3) Experiment 3**

Marker A: Precision Plus Protein Dual Color Standards (10-250kDa), Catalog #1610374;

1: Control; 2: si-NC; 3: si-MTOR; 4: Control + *Tg*Ag;5: si-NC + *Tg*Ag; 6: si-MTOR + *Tg*Ag;

Marker A: Precision Plus Protein Dual Color Standards (10-250kDa), Catalog #1610374;

1: Control; 2: si-NC; 3: si-MTOR; 4: Control + *Tg*Ag;5: si-NC + *Tg*Ag; 6: si-MTOR + *Tg*Ag;
